# Heterogeneity of HBV-Specific CD8^+^ T-Cell Failure: Implications for Immunotherapy

**DOI:** 10.3389/fimmu.2019.02240

**Published:** 2019-09-20

**Authors:** Kathrin Heim, Christoph Neumann-Haefelin, Robert Thimme, Maike Hofmann

**Affiliations:** ^1^Department of Medicine II, Faculty of Medicine, University Hospital Freiburg, University of Freiburg, Freiburg, Germany; ^2^Faculty of Biology, University of Freiburg, Freiburg, Germany

**Keywords:** chronic HBV infection, CD8^+^ T cells, exhaustion, T-cell heterogeneity, viral antigen

## Abstract

Chronic hepatitis B virus (HBV) infection is a major global health burden affecting around 257 million people worldwide. The consequences of chronic HBV infection include progressive liver damage, liver cirrhosis, and hepatocellular carcinoma. Although current direct antiviral therapies successfully lead to suppression of viral replication and deceleration of liver cirrhosis progression, these treatments are rarely curative and patients often require a life-long therapy. Based on the ability of the immune system to control HBV infection in at least a subset of patients, immunotherapeutic approaches are promising treatment options to achieve HBV cure. In particular, T cell-based therapies are of special interest since CD8^+^ T cells are not only capable to control HBV infection but also to eliminate HBV-infected cells. However, recent data show that the molecular mechanisms underlying CD8^+^ T-cell failure in chronic HBV infection depend on the targeted antigen and thus different strategies to improve the HBV-specific CD8^+^ T-cell response are required. Here, we review the current knowledge about the heterogeneity of impaired HBV-specific T-cell populations and the potential consequences for T cell-based immunotherapeutic approaches in HBV cure.

## Introduction

Infection with Hepatitis B virus (HBV) represents one of the major causes of morbidity and mortality worldwide. Despite the availability of a prophylactic vaccine for over 30 years, the number of infections remains dramatically high. Approximately, 257 million people globally suffer from chronic HBV infection (1). Current antiviral treatments such as nucleos(t)ide analogous (NUCs) can effectively inhibit HBV polymerase activity and decelerate the disease progression (2). However, there is a low prevalence of HBs antigen loss and anti-HBs seroconversion in patients undergoing NUC therapies and therefore a continuous clinical follow-up is necessary (3). In the clinical setting, HBs antigen persistence is a key marker for the diagnosis of chronic HBV infection. Anti-HBs seroconversion in turn is a marker for sustained viral control by the cellular and humoral immune response and thus is defined as “functional cure”. Identification of new therapeutic targets and development of additional therapeutic approaches are urgently needed. Antibody-mediated depletion studies in acutely HBV infected chimpanzees highlighted HBV-specific CD8^+^ T cells as the main effector cells that contribute to immunological control. In fact, in this study, a prolonged viremia was observed until the reappearance of HBV-specific CD8^+^ T cells in the blood and liver (4). In contrast, the development of a persistent HBV infection is associated with a compromised HBV-specific CD8^+^ T-cell response (5). The mechanisms underlying the impaired HBV-specific CD8^+^ T-cell dysfunction are still not completely understood. Recently, it was shown that the dysfunctional HBV-specific CD8^+^ T cells differ with respect to the targeted HBV antigens. Therefore, different strategies may be required to improve the HBV-specific CD8^+^ T-cell response. In this article, we focus on the heterogeneity of HBV-specific CD8^+^ T cells and the potential for reinvigoration of these populations during chronic HBV infection.

## Mechanisms of T-Cell Exhaustion in Chronic HBV Infection

Chronic HBV infection is associated with an impaired HBV-specific CD8^+^ T-cell response. Although the HBV-specific CD8^+^ T-cell response is initially induced, several phenotypic and functional deficiencies can be observed under the conditions of antigen persistence. These so-called exhausted CD8^+^ T cells represent a unique immune cell differentiation stage that was first described in mice chronically infected with the lymphocytic choriomeningitis virus (LCMV). In chronic HBV, early studies analyzing HBV-specific CD8^+^ T cells during persistent HBV infection also showed an compromised functionality of HBV-specific CD8^+^ T cells including reduced production of the antiviral cytokine interferon γ (IFNγ) and the immunomodulatory cytokine interleukin 2 (IL-2), as well as the loss of cytotoxicity and impaired proliferative capacities (6–8). Further studies showed that these functionally exhausted HBV-specific CD8^+^ T cells displayed a high expression of multiple inhibitory receptors like programmed cell death protein 1 (PD1), cytotoxic T-lymphocyte-associated protein 4 (CTLA-4), lymphocyte-activation gene 3 (LAG3), T-cell immunoglobulin mucin domain-3 (TIM3), CD244/2B4, or CD160. The co-expression of these markers contributes to T-cell dysfunction by excessive T-cell suppression (6, 9–14). Moreover, HBV-specific CD8^+^ T cells are characterized by an impaired T-cell homeostasis. In particular, the intracellular expression of the pro-apoptotic protein BCL2-interacting mediator (BIM) has been shown to be increased in chronically HBV-infected patients and to drive the decline of HBV-specific CD8^+^ effector T cells (15). A specific transcriptional profile has also been ascribed to the exhausted HBV-specific CD8^+^ T-cell phenotype (8, 16). In fact, the expression patterns of T-bet and Eomesodermin (Eomes) have been shown to tightly regulate differentiation of exhausted HBV-specific CD8^+^ T cells. Specifically, a dysregulated T-bet expression was found in exhausted HBV-specific CD8^+^ T cells, whereas Eomes was shown to compensate for the lack of T-bet (8). Recently, *ex vivo* transcriptome analysis of exhausted HBV-specific CD8^+^ T cells in chronically infected patients also revealed substantial mitochondrial dysfunction and impaired metabolism (16). These mitochondrial alterations contribute to the functional exhaustion in these patients (16, 17). Noteworthy, *in vitro* manipulation reinvigorated the antiviral activity of exhausted HBV-specific CD8^+^ T cells in short-term cultures. In these experiments, the addition of mitochondrion-targeted antioxidants or cytokines partly restored the cytokine production of these cells (16, 17).

Although excessive antigen triggering seems to be a main driver of T-cell exhaustion, several other factors may also play an important role (18). These include limited CD4^+^ T-cell help (19–23), the induction of suppression by regulatory T cells (Tregs) (24–27) and an immunosuppressive liver environment which is also characterized by the action of immunosuppressive cytokines such as IL-10 and transforming growth factor β (TGFβ) (23, 28).

Taken together, HBV-specific CD8^+^ T cells clearly show phenotypic and functional evidence of T-cell exhaustion in chronically infected patients. Noteworthy, recent studies demonstrated that exhausted CD8^+^ T cells do not represent a homogeneous T-cell population but are rather heterogeneous in phenotype and function.

## T-Cell Heterogeneity—Lessons From LCMV Mouse Model

**Figure 1:**
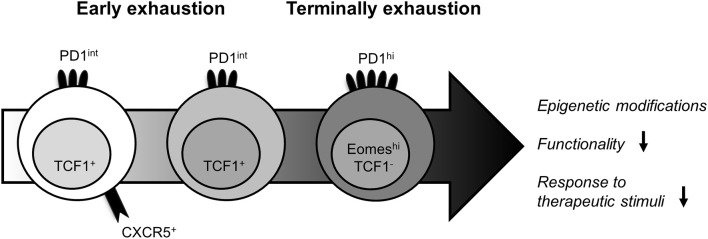
Heterogeneity of exhausted LCMV-specific CD8^+^ T cells. The identification of functionally distinct T-cell subpopulations within exhausted LCMV-specific CD8^+^ T cells has enabled the definition of their lineage dynamics. Despite the epigenetic fingerprint of T-cell exhaustion, the expression patterns of several phenotypical and transcriptional markers can discriminate between less exhausted and terminally exhausted LCMV-specific CD8^+^ T cells. While the early differentiated LCMV-specific CD8^+^ T-cell subpopulation is defined by co-expression of PD1, CXCR5, and TCF1 and provides a strong response to therapeutic stimuli, the more differentiated PD1^int^ TCF1^+^ LCMV-specific CD8^+^ T-cell subpopulation still harbors some effector function. In contrast, the terminally exhausted PD1^hi^, Eomes^hi^ LCMV-specific CD8^+^ T-cell population exhibit a more severely impaired functionality and is unresponsiveness to immunotherapies.

The LCMV mouse model first strongly contributed to dismiss the initial view about exhausted T cells to be a homogeneous dysfunctional population. Early studies have reported different subsets of exhausted LCMV-specific CD8^+^ T cells with distinct phenotypic and functional characteristics (Figure 1). The classification of these subsets is based on distinct expression patterns of the inhibitory receptors PD1 and CD44. In fact, two distinct exhausted LCMV-specific CD8^+^ T-cell subpopulations can be distinguished: the less functionally exhausted PD1^int^CD44^hi^ T-cell subset and the terminally exhausted PD1^hi^CD44^int^ counterpart (29, 30). Subsequently, by studying co-expression of the two T-box transcription factors T-bet and Eomes (31), it could be shown that the PD1^int^ T-cell subset was largely T-bet^hi^ and Eomes^lo^. This exhausted CD8^+^ T-cell subset functions as a progenitor population with improved proliferative capacity and cytokine production. In contrast, the terminally exhausted PD1^hi^ T-cell population has a quite unique expression pattern with a particularly high expression of Eomes and low expression of T-bet. Interestingly, some functionality was also retained from this PD1^hi^T-bet^int^Eomes^hi^ T-cell subset indicating that both exhausted CD8^+^ T-cell subsets are required to sustain viral control (31). Additional studies provided further evidence that these two exhausted CD8^+^ T-cell subsets are in a progenitor/progeny relationship. For example, the transcription factor T-cell factor 1 (TCF1) plays a central role (32, 33), as it is important for the establishment of CD8^+^ T-cell memory and for T-cell proliferation (34). Thereby, TCF1^+^PD1^int^ LCMV-specific CD8^+^ T cells represent a circulating T-cell subpopulation that sustains the LCMV-specific CD8^+^ T-cell pool during chronic viral infection (32, 33). Additionally, in lymphoid tissue, a population of chemokine receptor CXCR5 expressing TCF1^+^PD1^int^ LCMV-specific CD8^+^ T cells has been described that gives rise to the terminally exhausted T-cell pool in the periphery (35, 36). Overall, these combined findings revealed the functional T-cell heterogeneity within exhausted LCMV-specific CD8^+^ T cells. The biological importance of this T-cell heterogeneity in chronic infections was demonstrated by immunotherapeutic interventions, where the proliferative burst upon PD1 pathway blockade was almost exclusively restricted to the less differentiated progenitor/memory-like populations. In contrast, the terminally differentiated subset of exhausted LCMV-specific CD8^+^ T cells showed only a slight improvement in the T-cell response to PD1 pathway blockade that was associated with protective immunity (29, 31, 35, 36). However, PD1 pathway blockade does not fully restore exhausted CD8^+^ T cells due to an epigenetic imprinting of T-cell exhaustion (37–39). In fact, exhausted LCMV-specific CD8^+^ T cells differ from effector and memory CD8^+^ T cells by ~6,000 open chromatin regions. The comprehensive characterization of the genomic profile revealed significant alterations in the expression of genes encoding inhibitory receptors as well as transcription factors and genes controlling TCR signaling pathways, costimulatory and cytokine signaling, and cellular metabolism (38, 39). Furthermore, in several recent studies, the HMG box transcription factor TOX was identified as master regulator of T-cell exhaustion (40–42). In particular, a robust expression of TOX induces the fate commitment of an exhausted and dysfunctional phenotype in CD8^+^ T cells by driving epigenetic remodeling events at key gene loci (40, 41). Of note, despite this TOX-mediated epigenetic fingerprint of T-cell exhaustion, there are still differences in the epigenetic landscape of TCF1^−^PD1^hi^ vs. TCF1^+^PD1^int^ cells pointing toward stem-cell like characteristics of the latter subset within the exhausted LCMV-specific CD8^+^ T-cell population (43). In sum, these data clearly show that CD8^+^ T-cell exhaustion represents a distinct and stable differentiation lineage comprising different subsets that respond differently to therapeutic stimuli.

## Heterogeneity of Exhausted HBV-Specific CD8^+^ T Cells

**Figure 2:**
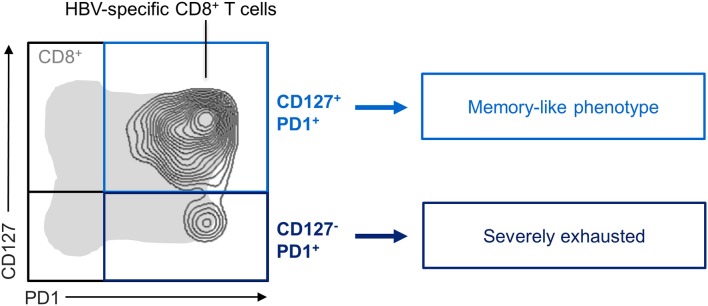
Exhausted HBV-specific CD8^+^ T-cell subsets. CD127/PD1 co-expression analysis of exhausted HBV-specific CD8^+^ T cells showed the existence of distinct subsets. While HBV-specific CD8^+^ T cells were predominant in the CD127^+^PD1^+^ memory-like subset, the more severely exhausted CD127^−^PD1^+^ subset was also found in a small proportion.

Recently, the co-existence of heterogeneous and distinctly differentiated exhausted virus-specific CD8^+^ T-cell subsets in human chronic viral infections such as human immunodeficiency virus (HIV) (44, 45) and chronic Hepatitis C virus (HCV) infection (46) have also been reported. Specifically, HCV-specific CD8^+^ T cells can be divided in distinct subsets based on the CD127/PD1 co-expression patterns. The CD127^−^PD1^hi^ HCV-specific CD8^+^ T-cell subpopulation clearly showed hallmarks of terminal differentiation, such as high levels of inhibitory receptors, indicating severe exhaustion in this subset. In contrast, the CD127^+^PD1^+^ HCV-specific CD8^+^ T-cell subset remarkably shared phenotypic and molecular properties with conventional memory CD8^+^ T cells. However, this subset also displayed characteristics of T-cell exhaustion such as an expression of PD1 and low functionality in terms of proliferation and cytokine production. Therefore, this population is referred to as memory-like (46). In HBV, previous studies on the phenotype of HBV-specific CD8^+^ T cells were often hampered by the low frequency of CD8^+^ T cells present in the peripheral blood of chronically HBV-infected patients (14). Recently, we have deployed pMHC I tetramer-based magnetic bead enrichment approaches to increase the number of detectable HBV-specific CD8^+^ T cells and thereby improving the potential for analyzing their phenotype, function, and subset distribution. By using this approach, we were able to detect HBV-specific CD8^+^ T cells in the majority of chronically HBV-infected patients with low viral load. Subsequently performed in-depth analyses revealed that HBV-specific CD8^+^ T-cell populations are indeed also heterogeneous on a subset level (Figure 2). Based on the CD127/PD1 co-expression analyses, we were able to ascribe the existence of distinct HBV-specific CD8^+^ T-cell subsets including less differentiated memory-like CD127^+^PD1^+^ and terminally exhausted CD127^−^PD1^+^ subpopulations. Interestingly, in contrast to HCV-specific CD8^+^ T cells, the memory-like CD127^+^PD1^+^ subset predominates in chronically HBV-infected patients with low viral load. This subset is further defined by markers characteristic for memory T cells like BCL2 and TCF1. Importantly, in contrast to conventional memory CD8^+^ T cells, HBV-specific CD8^+^ T cells also uniformly express PD1. Altogether, HBV-specific CD8^+^ T cells exhibit a distinct subset distribution in the setting of chronic HBV infection (47).

Heterogeneity of HBV-specific CD8^+^ T cells is also existent on the level of the targeted antigens. Indeed, immunological characterization of transgenic mice has already shown a hierarchy of dominant and subdominant HBV antigens with various frequencies and antiviral activity (48–50). Recently, studies in chronically HBV-infected patients also observed different properties of HBV-specific CD8^+^ T cells targeting different HLA-A^*^02:01 restricted epitopes located in the core (core_18−27_: FLPSDFFPSV), envelope (env_183−191_: FLLTRILTI), and polymerase (pol_455−463_: GLSRYVARL) proteins (Table 1). First, the frequencies of HBV-specific CD8^+^ T cells targeting the different epitopes varied significantly. Core_18_-specific CD8^+^ T cells were present in a higher frequency compared to pol_455_-specific CD8^+^ T cells, whereas env_183_-specific CD8^+^ T cells were rarely detectable in patients with chronic HBV infection (47, 51). The low frequency of env_183_-specific CD8^+^ T-cell responses was generally related to the high levels of HBs antigen and is thus most likely caused by deletion as a consequence of hyperactivation. Second, differences within the CD127/PD1-based subset distribution were observed between core_18_- and pol_455_-specific CD8^+^ T cells in chronically HBV-infected patients. Precisely, pol_455_-specific CD8^+^ T cells showed a diminished proportion of the memory-like CD127^+^PD1^+^ subset compared to core_18_-specific CD8^+^ T cells (47). This finding together with the distinct expression of killer cell lectin like receptor G1 (KLRG1), Eomes and CD38 on pol_455_-specific CD8^+^ T cells reflected a higher degree of terminal T-cell exhaustion compared with core_18_-specific CD8^+^ T cells (47, 51). The different exhaustion profile of both HBV-specific CD8^+^ T-cell subpopulations was further underpinned by the functional analyses revealing decreased expansion capacity of pol_455_-specific CD8^+^ T cells which was linked to a dysregulated TCF1/BCL2 expression (47). Thus, these findings give a first hint that T-cell failure of HBV-specific CD8^+^ T-cell populations may occur due to different molecular mechanisms. Additionally, in both studies (47, 51), phenotypic and functional differences of HBV-specific CD8^+^ T cells targeting core vs. polymerase epitopes were also evident in the context of non-HLA-A^*^02 alleles (HLA-A^*^01:01: core_30−38_: LLDTASALY; HLA-A^*^11:01: core_88−96_: YVNVNMGLK; core_141−150:_ STLPETVVRR; HLA-A24:02: core_117−125_: EYLVSFGVW, pol_756−764_: KYTSFPWLL; HLA-B^*^08:01: core_123−130_: GLKILQLL; HLA-B^*^35:01: core_19−27_: LPSDFFPSV, pol_173−181_: SPYSWEQEL; HLA-B51:01: core_19−27_: LPSDFFPSV; HLA-B^*^40:01: pol_40−48_: AEDLNLGNL) indicating an antigen-related phenomena. In line with this observation, another recent study also highlighted different exhaustion profiles of HBV-specific CD8^+^ T cells targeting different HLA-A^*^11:01 restricted epitopes within the core and polymerase antigens (core_169−179_: STLPETAVVRR and pol_387−396_: LVVDFSQFSR) (52). Furthermore, Cheng et al. showed that HBV-specific CD8^+^ T-cell heterogeneity is also associated with the status of HBV infection (52). Still, a more comprehensive study is required to precisely dissect the impact of epitope, HLA-restriction, antigen, and disease status on HBV-specific CD8^+^ T-cell heterogeneity. Another open point that needs to be addressed in future studies is the HBV-specific CD8^+^ T-cell heterogeneity within the liver since current studies have focused on circulating lymphocytes within the blood and thus the effect of a possible compartmentalization has not been taken into account. This knowledge might be of particular importance for immunotherapeutic approaches since different strategies have potentially to be applied to boost the heterogeneous HBV-specific CD8^+^ T-cell populations.

**Table 1:** Different facets of exhausted HBV-specific CD8^+^ T cells targeting different antigens.

	**Core**	**Pol**
Frequency	+++	++
Memory-like subset	TCF1↑ BCL2↑	TCF1↑ BCL2↓
Expansion capacity	↑	↓
Degree of T-cell exhaustion	↓	↑

**Figure 3:**
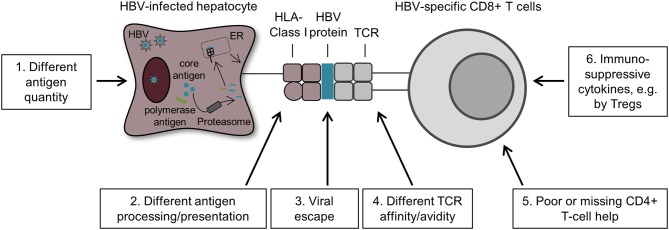
Possible mechanisms that drive HBV-specific CD8^+^ T-cell heterogeneity. First, HBV-infected hepatocytes produced varying amounts of HBV antigens resulting in different quantities of HBV peptide/HLA complexes on these cells. This difference may modulate the heterogeneity of HBV-specific CD8^+^ T cells targeting different antigens (1). Second, HBV antigen processing and presentation may also impact the phenotype and function of core_18_- and pol_455_-specific CD8^+^ T cells. In fact, HBV core antigens are secreted in high quantities by HBV-infected hepatocytes and are therefore more likely cross-presented by antigen-presenting cells, whereas the low amount of HBV polymerase antigens are primarily presented by HBV-infected hepatocytes (2). Third, the phenomenon of viral escape also affects antigen recognition by HBV-specific CD8^+^ T cells and thus may also contribute to HBV-specific CD8^+^ T-cell heterogeneity (3). Moreover, several other factors are also likely to promote HBV-specific CD8^+^ T-cell heterogeneity such as TCR affinity/avidity (4), a poor or missing CD4^+^ T-cell help (5) and the presence of immunosuppressive cytokines produced by Tregs and DCs (6).

The mechanisms responsible for different exhaustion profiles of circulating HBV-specific CD8^+^ T cells specific for distinct HBV antigens are still unclear (Figure 3). Nevertheless, one hypothesis is that different quantities of HBV antigens produced in infected hepatocytes may play a role in this process. In particular, in the infected liver, HBc antigen is present in higher amounts compared to polymerase antigen (53). This seems contradictory to the less exhausted phenotype of core_18_- vs. pol_455_-specific CD8^+^ T cells (47, 51). Since antigen recognition is one of the main drivers of T-cell exhaustion, one possible explanation for this phenomenon is that the processing and presentation of core peptides is altered which may affect the core_18_-specific CD8^+^ T-cell activation. Thus, HBV antigen presentation rather than solely the antigen level may contribute to HBV-specific CD8^+^ T-cell heterogeneity. A recent study showed that HBV antigen presentation is also heterogeneous within an HBV-infected liver. Indeed, HBV-infected hepatocytes expressed antigens in different quantities and localizations and from different sources resulting in a non-uniformal HLA class I/HBV epitope presentation in the liver and thereby triggering different level of HBV-specific CD8^+^ T-cell activation (54). Moreover, antigens produced and secreted in high quantities by HBV-infected hepatocytes such as HBc antigen may be presented either by hepatocytes themselves or cross-presented by other professional antigen-presenting cells (55). Thus, the different initial CD8^+^ T-cell priming process along with differences in quality and dynamics of epitope processing may lead to different antiviral efficacies of HBV-specific CD8^+^ T cells targeting different specificities. Additionally, viral escape also affects antigen recognition and thus may contribute to HBV-specific CD8^+^ T-cell heterogeneity. Of note, in contrast to the previous studies on escaped epitopes in chronic HCV (56), the presence of HBV sequence variations within the core_18_ epitope did not alter the functional or phenotypic fingerprints of core_18_-specific CD8^+^ T cells at all (47, 51). Several other mechanisms probably may also favor HBV-specific CD8^+^ T-cell heterogeneity such as different TCR affinity/avidity, a poor or missing CD4^+^ T-cell help or immunosuppressive cytokines produced by Tregs and dendritic cells (DCs) (57). Hence, further studies are required to identify and dissect the determinants of HBV-specific CD8^+^ T-cell heterogeneity. Overall, these new findings showed that circulating HBV-specific CD8^+^ T cells are not equal and in fact are differentially characterized depending on their antigen specificity.

## Application of Exhausted HBV-Specific CD8^+^ T-Cell Heterogeneity to Immunotherapy

Since the discovery of T-cell exhaustion, researchers are investigating the potential for functional restoration of exhausted T-cell populations. Such approaches aim to restore an endogenous dysfunctional antiviral immune response. Indeed, great efforts have been made to boost HBV-specific CD8^+^ T-cell responses by blocking the interactions of inhibitory receptors with their ligands (e.g., PD1, CTLA-4, or CD244/2B4 pathway blockade). *In vitro* studies have shown an at least partial restoration of HBV-specific CD8^+^ T cells including an enhanced proliferative potential and increased cytokine production upon checkpoint pathway blockade (6, 9, 11–14). Recently, a pilot study performed in HBeAg negative chronically HBV-infected patients treated with nivolumab (PD-1 pathway blockade) showed a modest decline of HBsAg in most of the patients, while one patient even achieved a sustained HBs antigen loss and anti-HBs seroconversion (58). However, treatment of patients with advanced hepatocellular carcinoma and HBeAg negative chronic HBV infection with nivolumab in a phase 1/2 clinical trial had no effect on antiviral immune response and caused no anti-HBs seroconversion (59). The above reviewed recent research offers a novel perspective on the complexity of the T-cell response present in chronically HBV-infected patients that needs further investigation in light of immunotherapy, in particular with respect to responsiveness and antiviral efficacy. So far, it is still not understood which HBV-specific CD8^+^ T-cell subpopulation might endow with a better antiviral activity. The current findings suggest that core_18_-specific CD8^+^ T cells have a less impaired functionality than pol_455_-specific CD8^+^ T cells reflected by their mildly exhausted phenotype. Thus, this finding indicates that core_18_-specific CD8^+^ T cells may show an improved responsiveness to PD1 pathway blockade. In contrast, pol_455_-specific CD8^+^ T cells which exhibit a terminally exhausted phenotype may only poorly respond to PD1 pathway blockade as already described in the chronic LCMV mouse model (29, 31, 35, 36). However, reinvigoration of the pol_455_-specific CD8^+^ T-cell response might be an efficient approach to achieve HBV cure. It is known that the pol_455_-specific CD8^+^ T-cell response is particularly impaired in chronic HBV infection compared to acutely resolved infection, whereas core_18_-specific CD8^+^ T cells are similar in their expansion capacity in both HBV infection settings (47). Moreover, metabolic alterations of HBV-specific CD8^+^ T cells highlight the difficulty of boosting HBV-specific CD8^+^ T cells. Interestingly, a marked recovery of antiviral capacity was achieved by treating core_18_-specific CD8^+^ T cells with mitochondrion-targeted antioxidant compounds *in vitro* (16). Further studies are now needed to investigate the effect of metabolic reprogramming on pol_455_-specific CD8^+^ T-cell responses since the combination of compounds targeting both, the impaired metabolism together with checkpoint blockade, provides a promising option in HBV treatment. In light of the recent finding that TOX represents a master regulator of T-cell exhaustion (40–42), it is tempting to speculate that TOX-mediated epigenetic changes are also involved in HBV-specific CD8^+^ T-cell dysfunction. Hence, to boost the defective pol_455_-specific CD8^+^ T-cell response, combining the PD1 pathway blockade with epigenetic modifications might be necessary. Early studies on the manipulation of epigenetic pathways have shown promising results to overcome T-cell exhaustion in chronic viral infections. Indeed, treatment of exhausted LCMV-specific CD8^+^ T cells with either histone deacetylate inhibitors (60) or the blockade of *de novo* DNA methylation (61) rescued LCMV-specific CD8^+^ T cells from functional exhaustion. Thus, attempts to manipulate the epigenetic signature could have important clinical implications. However, such studies investigating therapeutic manipulations of epigenetic changes have not been performed yet in chronic viral hepatitis and probably face safety issues due to the broadly acting characteristics of currently available reagents. As a consequence of the terminally exhausted phenotype of pol_455_-specific CD8^+^ T cells and the complexity in restoring pol_455_-specific CD8^+^ T-cell immunity, another immunotherapeutic strategy is based on adoptive transfer of engineered T cells. Recently, it was demonstrated by Kah et al. that engineered T cells that transiently express HBV-specific T-cell receptors exert a great antiviral effect in HBV-infected humanized chimeric mice (62). The extreme rarity of env_183_-specific CD8^+^ T cells might be caused by the induction of T-cell tolerance which results in reduced T-cell expansion and elevated T-cell apoptosis (63). Thus, for env_183_-specific CD8^+^ T cells in chronic HBV patients, the usage of adoptive transfer is also implicated. However, the induction of novel env_183_-specific CD8^+^ T-cell responses by therapeutic vaccines should additionally be considered. So far, different therapeutic vaccines are already used in several clinical trials with limited effect on chronic HBV infection (64–67). In sum, the heterogeneity of HBV-specific CD8^+^ T cells suggest that HBV cure can probably not be achieved by only one single approach. Therefore, a combination of different immunotherapeutic interventions might be necessary to induce viral elimination or at least T cell-based control in chronic HBV infection.

## Conclusion

Exhausted T cells consist of functionally diverse T-cell subpopulations that co-exist during chronic HBV infection. These findings revealed that HBV-specific CD8^+^ T cells specific for different HBV proteins harbor distinct phenotypical and more importantly functional features in chronically HBV-infected patients. This knowledge allows specializing future immunotherapeutic approaches to target the specific T-cell subpopulation and its molecular pathway that is suitable for the desired kind of immunomodulation. However, the current state of immunotherapeutic treatments suggests that the task of controlling chronic HBV infection is quite difficult. Further characterization of the recently discovered HBV-specific CD8^+^ T-cell subpopulations are needed to uncover new molecular pathways that could be additionally targeted by immunotherapeutic interventions.

## Author Contributions

All authors listed have made a substantial, direct and intellectual contribution to the work, and approved it for publication.

### Conflict of Interest Statement

The authors declare that the research was conducted in the absence of any commercial or financial relationships that could be construed as a potential conflict of interest.
